# High geothermal heat flux in close proximity to the Northeast Greenland Ice Stream

**DOI:** 10.1038/s41598-018-19244-x

**Published:** 2018-01-22

**Authors:** Søren Rysgaard, Jørgen Bendtsen, John Mortensen, Mikael K. Sejr

**Affiliations:** 1 Arctic Research Centre, Department of Bioscience, Aarhus University, 8000 Aarhus, Denmark; 2 Greenland Climate Research Centre, Greenland Institute of Natural Resources, PO Box 570, 3900 Nuuk, Greenland; 3 Centre for Earth Observation Science, CHR Faculty of Environment Earth and Resources, University of Manitoba, 499 Wallace Building, Winnipeg, Manitoba R3T 2N2 Canada; 4 Climatelab, Symbion Science Park, Fruebjergvej 3, PO box 98, 2100 Copenhagen O, Denmark

## Abstract

The Greenland ice sheet (GIS) is losing mass at an increasing rate due to surface melt and flow acceleration in outlet glaciers. Currently, there is a large disagreement between observed and simulated ice flow, which may arise from inaccurate parameterization of basal motion, subglacial hydrology or geothermal heat sources. Recently it was suggested that there may be a hidden heat source beneath GIS caused by a higher than expected geothermal heat flux (GHF) from the Earth’s interior. Here we present the first direct measurements of GHF from beneath a deep fjord basin in Northeast Greenland. Temperature and salinity time series (2005–2015) in the deep stagnant basin water are used to quantify a GHF of 93 ± 21 mW m^−2^ which confirm previous indirect estimated values below GIS. A compilation of heat flux recordings from Greenland show the existence of geothermal heat sources beneath GIS and could explain high glacial ice speed areas such as the Northeast Greenland ice stream.

## Introduction

A recent notable increase in surface speed is observed in the northeastern region of Greenland originating from the center of GIS and exiting through the ice-stream outlet glaciers; Nioghalvfjersbræ, Zachariæ Isstrøm and Storstrømmen^1^. Velocities of ice streams are in general increasing and thereby increasing mass loss – but changes in ice-stream dynamics are not well understood and thus cannot be predicted^1,2^. Model studies show that GHF from the Earth’s interior influence the thermal structure of GIS and the distribution of basal meltwater^3^. Although direct measurements of GHF in Greenland are nearly absent, model estimates suggest a regional difference with reported values ranging from 20 to 40 mW m^−2^ in the south to 140 mW m^−2^ in the central northern Greenland^4–6^. Locally, high melt rates indicate GHF 15 to 30 times higher than continental background values^5^. This could strongly influence basal hydrology, sediment deformation and discharge, subglacial lakes and basal erosion forms^7^. Geothermal springs with source water temperatures above 0 °C have been found all over Greenland, especially around Disko Island in West Greenland, where several thousands of such springs have been identified^8^. However, the hottest springs, with source water temperatures of 55-62 °C are found in East Greenland at a number of locations north and south of Scoresbysund^9,10^. East Greenland constitutes a volcanic rifted margin that forms part of the Tertiary North Atlantic Igneous Province^11^. The province developed before, during and after continental breakup, which took place in the earliest Eocene at around 55 Ma. The East Greenland margin extends for nearly 2000 km between 60°N and the Greenland Fracture Zone around 78°N^12^.

Recently, two large hydrothermal vent complexes (>28 000 km^2^) have been discovered on the Northeast Greenland shelf^13^. These vents are caused by overpressure build-up associated with boiling of pore fluids and rock reactions^14^ resulting in fracturing and venting of fluids and heat to the seafloor. In general, shallow water vents have lower temperature due to the interior heat source and some may have a meteoric water source in combination with seawater^15^.

**Figure 1:**
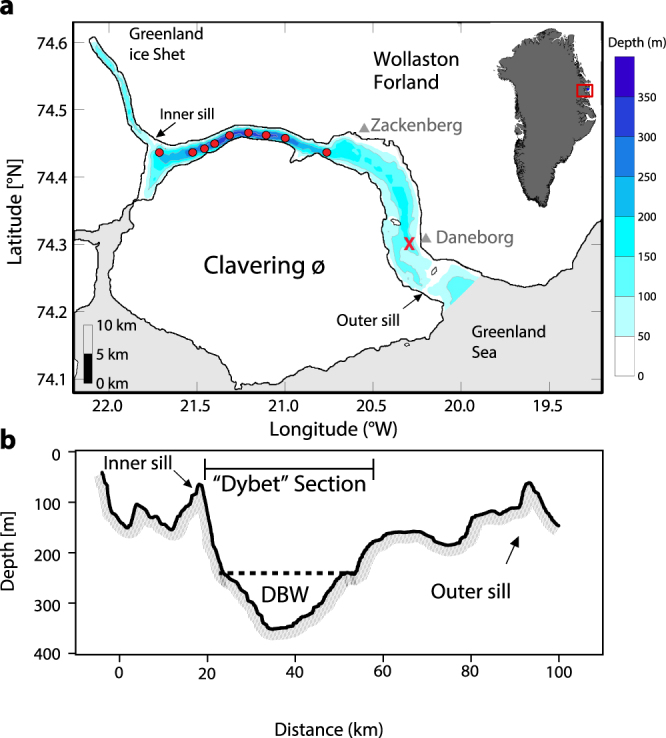
Study site in the Young Sound-Tyrolerfjord, Northeast Greenland. (**a**) The fjord is 90 km long and connects the Greenland ice sheet with the Greenland Sea. Red ‘X’ marks the continuous mooring recordings at 65 m. Locations for temperature and salinity measurements are indicated with red dots. (**b)** Bathymetry of the fjord showing the deep basin water and the two sills. DBW refers to “Dybet” bottom water. (Figure was modified from Rysgaard, S. *et al*.^18^ and generated with Illustrator v15.01.0, www.adobe.com).

In this study, we analyze hydrographic records from 2005–2015 from the deepest basin of the Young Sound-Tyrolerfjord system in Northeast Greenland (74°N) and present evidence for a large GHF in the area (Fig. 1). The fjord is a sill fjord with a deep basin and penetrates ~90 km inland where it is connected to GIS by several land terminating glaciers^16,17^. The relatively shallow outer sill (45 m depth) at its entrance limits exchange of seawater between the deep fjord basin and the adjacent shelf^18^. During summer, hydrographic conditions in the fjord are characterized by highly stratified surface waters and a relatively wide range of salinities (17 to 33.4) and temperatures (−1.6 °C to 8 °C)^19^, while winter conditions are weakly stratified with near ocean freezing temperatures (*c*. −1.8 °C) and a narrow salinity range (within 0.2 psu)^20^. Young Sound-Tyrolerfjord is connected to the East Greenland Current (EGC), which transports large amounts of freshwater from the Arctic Ocean via Fram Strait^21^. Polar Surface Water constitute the upper part of the EGC with salinities below 34.4 and temperatures less than 0 °C and relatively warm (0–2 °C) re-circulating Atlantic Water is found between 150 and 800 m depth at the continental slope^22^.

**Figure 2:**
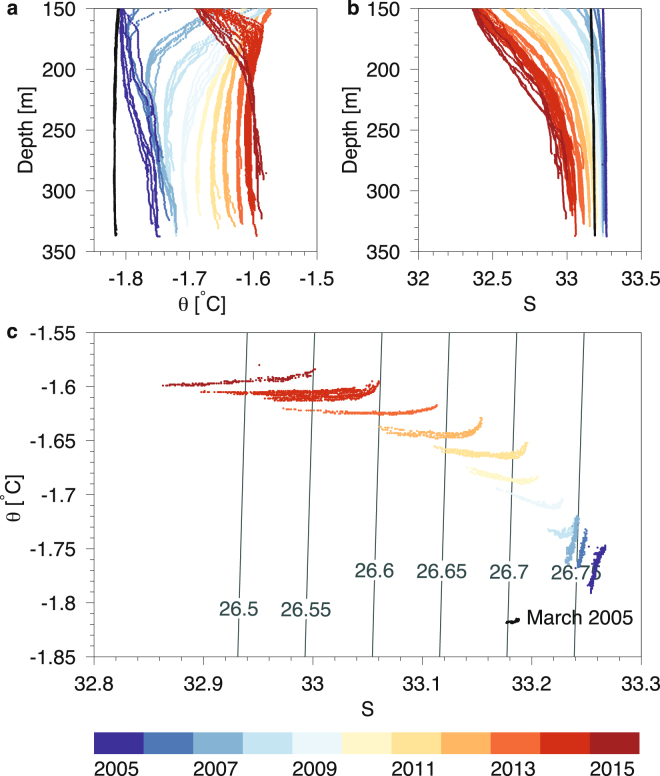
Potential temperature and salinity in the “Dybet” section. (**a)** Potential temperature (θ) and (**b**) salinity (S) versus depth (m) below 150 m and (**c**) corresponding θ-S diagram for depth levels below 240 m (isopycnals are shown in dark gray). Gradual color shading shows annual profiles between 2005 (blue) and 2015 (red). Profiles from March 2005 are shown in black.

On a sea ice expedition to the fjord in March 2005 we noticed that water temperatures started to increase in the deepest part of Tyrolerfjord from ~250 m depth toward the bottom in the deep basin and this temperature gradient has also been present in the subsequent measurements from this area since (Fig. 2). The heat source for this deep heating was analyzed further by considering all profiles of potential temperature (θ) and salinity (S) from the period 2005–2015 in the deepest part of the fjord, i.e. the “Dybet” section (see Methods, and Fig. 1). In March 2005 the water was characterized by near-freezing temperatures (<−1.8 °C) and relatively low salinities (<33.2). This low-density water mass (σ_θ_ = 26.71 kg m^−3^) was replaced by a more saline (S = 33.26) and slightly warmer (θ = −1.78 °C) and denser (26.76 kg m^−3^) water mass between March and August 2005 (Fig. 2a,b). The salinity increase toward the bottom was relatively small in August 2005 and the potential temperature increased from −1.82 °C at 150 m depth to −1.76 °C close to the bottom. Subsequently there were no indications of further major bottom water intrusions or deep convection in the period from 2006–2015 where θ and S below 240 m in average increased by 0.016 °C yr^−1^ and decreased by 0.03 psu per year, respectively. During this period the highest potential temperature below 250 m was observed close to the bottom in the “Dybet” section and this temperature increase was also manifested as a concave shape of the annual measurements in the θS-curve (Fig. 2c).

The annual temperature increase of bottom water below 240 m depth could either be explained by a gradual bottom water renewal in the fjord system or due to internal processes in the “Dybet” section, i.e. turbulent vertical mixing and GHF. Thus, the potential for gradual bottom water renewal was analyzed by comparing all profiles in the “Dybet” section from each year with corresponding profiles of coastal water masses outside the fjord (data not shown). The sill depth of 45 m is a barrier for bottom water exchange and to account for a potential isopycnal heave of ~15 m, we compared coastal water mass characteristics down to 60 m with the densities below 240 m depth in the “Dybet” section. Except for 2005, i.e. the year with a major bottom water renewal, all profiles showed that the density of water in the “Dybet” section below 240 m depth was higher than coastal water masses down to 60 m depth. However, all profiles from 2006–2015 were obtained during the first two weeks of August and, therefore, the density of the coastal water could potentially increase during winter and then lead to dense bottom water intrusions. A continuous mooring located in Young Sound close to the outer sill (see Fig. 1) measured temperature and salinity at 65 m depth i.e. ~80 m above the largest depth at the cross section of the fjord at this location and, therefore, bottom water inflow could occur without being detected at the mooring. However, measurements from the entire period show that the highest density and salinity occurred in late summer, and this support the conclusion above based on water mass analysis of measurements from July-September. Relatively large vertical θ and S-gradients in this period also indicated that deep convection did not penetrate below 150 m. In December 2013, a storm event created a coastal polynya at the entrance of the fjord that was maintained until March 2014^23^. During this period an enhanced surface transport was recorded out of the fjord and a layer between 40–140 m depth was flowing into the fjord and supplying the interior with cool and saline water from the polynya. This inflow layer gradually mixed with the deep basin water and explains the cooling seen of the upper basin water (i.e. above ~200 m) between 2013–2014 in the basin water (Fig. 2a). Therefore, based on water mass analysis of the deepest “Dybet” section below 240 m depth, we argue that there were no observations of bottom water renewal in the period 2006–2015.

**Figure 3:**
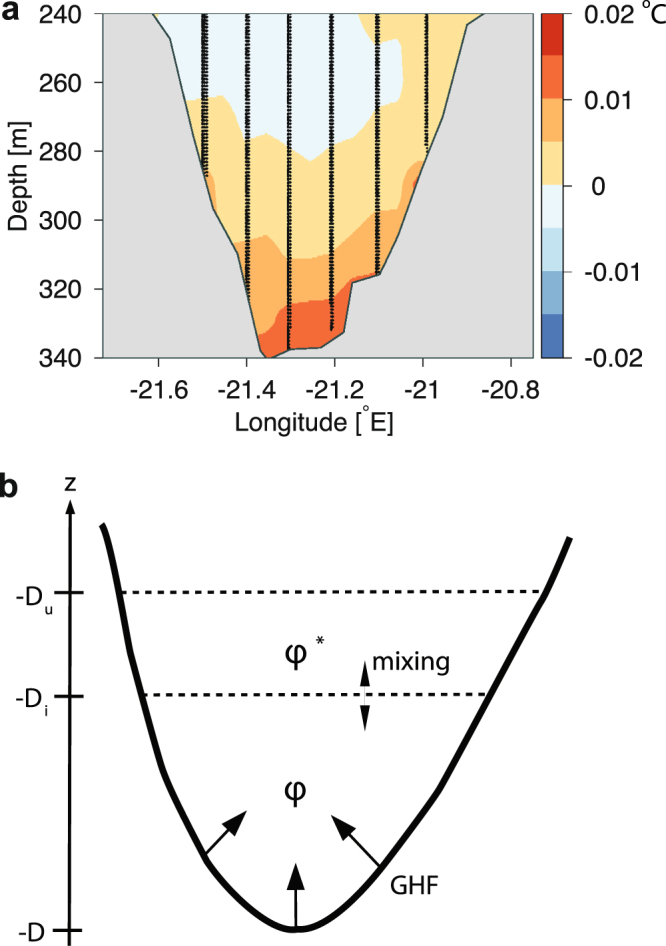
“Dybet” section temperature anomalies. (**a**) Potential temperature difference (°C) averaged in the period between 2005 and 2015. The annual potential temperature difference is defined by the difference between *in situ* potential temperature and the average value between 260 and 280 m, i.e. the depth interval of the deep temperature minimum. (**b**) Box model of the deepest part of the “Dybet” section.

Large vertical turbulent mixing has been observed in other deep fjords around Greenland^24^ and vertical mixing could also explain the observed θ and S-changes in “Dybet”. In addition, the near-bottom temperature increase could be due to a large GHF, and this phenomenon was analyzed further from the annual CTD-profiles in the period. An annual potential temperature anomaly (Δθ = θ − <θ>) was defined by considering all profiles of θ in the Dybet section below 240 m in relation to the annually averaged θ of all profiles between 260–280 m (<θ>), i.e. the depth range with the observed temperature minimum. The spatial distribution of Δθ for each year showed that maximum temperature anomalies were always found close to the bottom and the averaged distribution for the period 2006–2015 also showed a significant temperature increase of up to 0.02 °C (Fig. 3a). Thus, the gradual temperature increase during 2006–2015 and the spatial distribution of Δθ strongly indicate that bottom water temperatures increase due to a large GHF in the “Dybet” section. Therefore, we assume that vertical turbulent mixing and GHF are the primary processes behind the observed salinity and temperature change.

The GHF was calculated from a simple box model of the deepest part of the Dybet section (Fig. 3b). Temporal change of a tracer (φ, e.g S, θ or the heat content H) below the interface depth (D_i_ = 240 m) was assumed to be due to vertical turbulent mixing in the period 2006–2015 and, thereby, described by the one-dimensional conservation equation:

1 $$\frac{\partial \phi }{\partial t}=\frac{\partial }{\partial z}{k}_{v}\frac{\partial \phi }{\partial z}$$

where *t* and *z* is time and the vertical coordinate, respectively, and *k*_*v*_ is the vertical turbulent diffusion coefficient. Furthermore, it was assumed that the concentration is constant below D_i_, in general accordance with observations (i.e. the vertical gradients below 240 m are relatively small compared with the annual change, Fig. 2). Vertical exchange across the interface at D_i_ was calculated from the observed annual change in salinity.

Thereby, heating due to GHF could be calculated as: GHF = F_Htot_ − F_Hmix_, where F_Htot_ explains the total annual heat change and F_Hmix_ contains the contribution from vertical mixing of heat across the interface D_i_ (Fig. 3).

**Figure 4:**
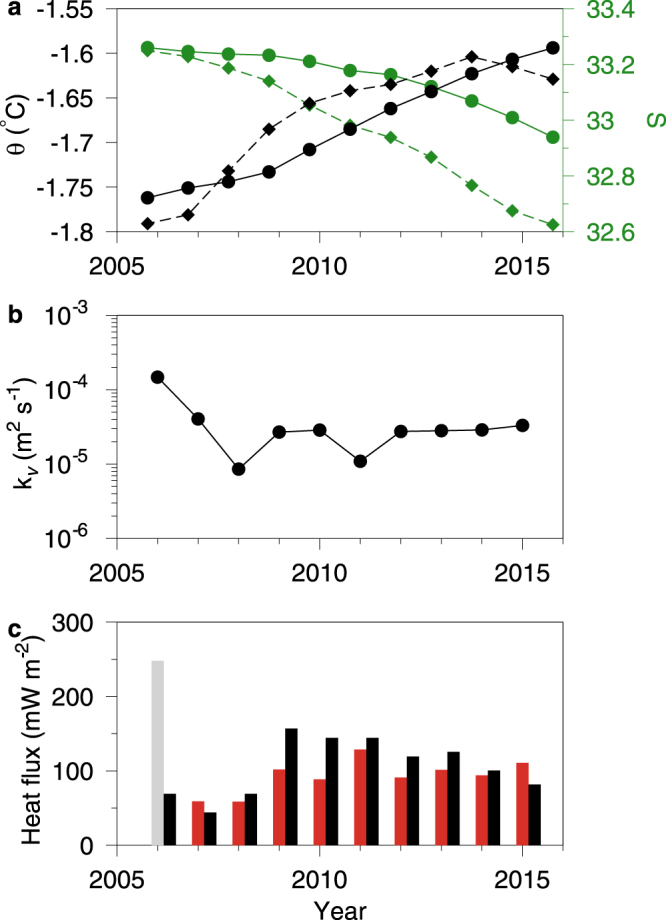
Box model results and heat fluxes. (**a)** Averaged observed potential temperature (black) and salinity (green) in “Dybet” below 240 m depth (solid lines and bullets), and from 140–240 m depth (dotted lines and diamonds) between 2005 to 2015. (**b**) Calculated turbulent diffusion coefficients. (**c**) GHF (red bars) and the total energy flux (black bars) including vertical mixing of heat. Value from 2005 (grey bar) was affected by bottom water renewal.

Overall, there has been a gradual warming of the stagnant deep basin bottom water below 240 m depth of 0.017 °C yr^−1^ from 2005 to 2015 (Fig. 4a). Salinity decreased by 0.03 yr^−1^ (Fig. 4a) and the calculated diffusion coefficient (Eq. 3) ranged between 10^–5^ to 10^−4^ m^2^ s^−1^ in the period 2006–2015 (Fig. 4b). These values are similar to observed values in the open Greenland Sea^25^ and below mixing intensities in high-tidal influenced Greenland fjords^24^. Assuming that the total temperature change below 240 m depth originates from the bedrock heat flux below the deep basin, this represents a total heat flux of 110 ± 36 mW m^−2^ in the period 2006–2015 (Fig. 4c). If we account for the concurrent freshening of the bottom water (0.03 yr^−1^) due to vertical turbulent mixing, the GHF then adjusts to 93 ± 21 mW m^−2^. This is much higher than previous reported values ranging from 20 to 40 mW m^−2^ in southern Greenland^4,6^, but confirm the indirect estimated values of 88–140 mW m^−2^ reported for the central northern Greenland^5,6,26^. We consider the inter-annual variability of the calculated GHF to be explained by uncertainties in estimating the spatial and temporal gradients from observations in Eq. 4 and, therefore, in accordance with a constant GHF of ~93 mW m^−2^. It should be noted that this heat flux and the observed salinity decrease during the period could be explained by a deep meteoric water source. However, to account for the observed temperature and salinity change this would imply a relatively high out-flowing temperature of more than 16 °C and a corresponding volume flow of less than 0.03 m^3^ s^−1^. A more likely out-flowing temperature range of ~0 °C, corresponding to glacial meltwater, would imply a volume flux of more than 1 m^3^ s^−1^ and lead to a salinity change of ~50 times larger than observed. Therefore, the GHF can either be explained as a conductive heat flux or as due to a weakly outflowing and warm deep (>16 °C) meteoric water source and, in this case, the calculated GHF would be a lower bound because less of the temperature increase in Dybet would be due to vertical turbulent transports in the water column.

**Figure 5:**
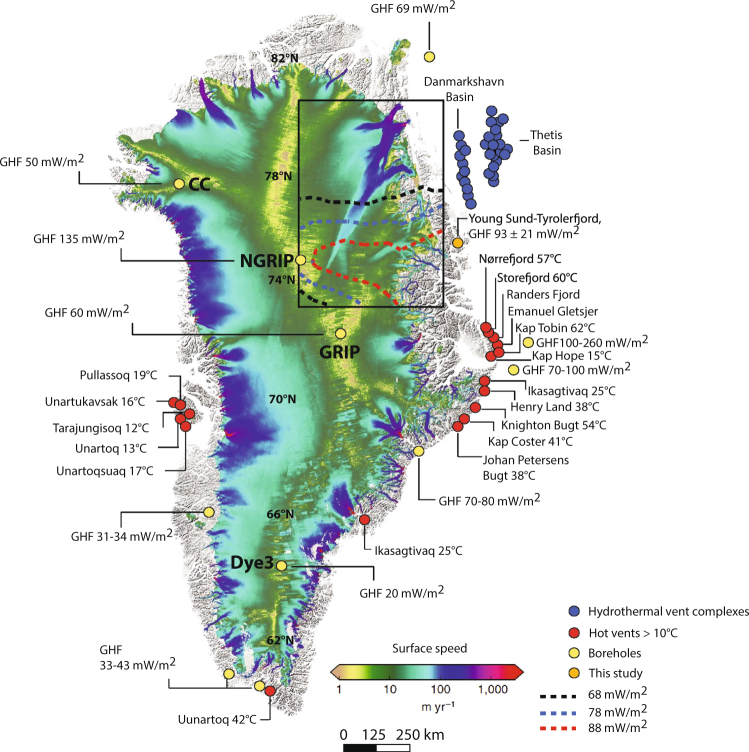
Geothermal vents localities and ice surface speeds (2008–2009) for Greenland. Geothermal vent localities on land with temperatures >10 °C^8–10^, Boreholes^26^, hydrothermal vent complexes offshore^13^ and present study. Reconstructed geothermal anomalies (contours in inserted box)^26^. Ice drilling localities are indicated by CC, NGRIP, GRIP and Dye3. (Figure with ice surface speeds was modified from Aschwanden, A. *et al*.^1^ and generated with Illustrator v15.01.0, www.adobe.com).

Combining our measurements with other sites of reported geothermal activity around Greenland clearly shows that East Greenland is a hotspot (Fig. 5). Several hot vents exist on land, with temperatures ranging from 15 to 62 °C and a tendency to increase northeastwards. In addition, GHF of 70–80 mW m^−2^ is reported near the Kangerdlugssuaq glacier (68°N)^26^; 70 to 260 mW m^−2^ is reported off the entrance of Scoresbysund fjord (higher on the northern side)^26^; 93 mW m^−2^ from the Young Sound-Tyrolerfjord (74°N, this study) and 69 mW m^−2^ off Nioghalvfjerdsbræ (79°N)^26^. It is notable that one of the largest landmasses in Greenland with glaciers melted far inland is situated exactly in this geothermal hotspot area between Scoresbysund fjord and Young Sound Tyrolerfjord. Elevated GHF providing heat for basal melt and, thereby, increasing glacier sliding over the bedrock could be a contributing factor although local ice geometrical settings, subglacial hydrology and the mechanical properties of the ice-bedrock interface are also important modulating factors.

High glacier surface speeds and negative mass balance are also observed near other hot vents in West Greenland such as the Jakobshavn Isbræ (69°N) near Disko Island; the Narssaq bræ, Valhaltinde glacier, Nordbo glacier and Qassimiut ice lobe in South Greenland all show negative ice mass balances near the hot vent in Uunartoq (60°N); in East Greenland the Helheim glacier, Isertoq and Mittivakkat glaciers located near Ikasagitavaq (66°N) are losing mass, and high ice speed is also observed at the Kangerdlugssuaq glacier where a GHF of 70–80 mW m^−2^ has been recorded (68°N)^1,16^ (Fig. 5). A reconstruction of the GHF across central northern Greenland was presented in a recent study^26^ (inserted box^26^ in Fig. 5). Their study was based on coupled three-dimensional climate-forced models of GIS and the underlying lithosphere and various validation data. A GHF anomaly of up to 88 mW m^−2^ was modeled to be located at the head of the 750-km-long Northeast Greenland ice stream. In addition, a GHF value of 135 mW m^−2^ has been reported at NGRIP^6^. Our recorded GHF of 93 mW m^−2^ in the Young Sound-Tyrolerfjord is located in this GHF hotspot and supports their model results. Hence, this part of Greenland may play a role for the rapid basal melt located at the head of the Northeastern Greenland ice stream and its high ice speeds. In addition, the newly discovered 52 hydrothermal vent complexes^13^, some of which reach up to 11 km in diameter, in the Danmarkshavn Basin and in the Thetis Basin is located just outside the ice-stream outlet glaciers; Nioghalvfjersbræ, Zachariæ Isstrøm and Storstrømmen (Fig. 5). These complexes are formed from hot intrusions (*c*. 1200 °C) at 1–2 km depth in the Thetis Basin and >3 km depth in the Danmarkshavn Basin. Hence, this accumulated evidence point to active geothermal activity in the northeastern corner of Greenland and indicate that geothermal heat source may exist below the center and northeastern part of GIS. This heat source may explain the origin of the Northeast Greenland ice stream and other areas with high ice stream speed.

## Methods

CTD (conductivity, temperature and depth) observations in the water column were made using a SBE 19 + instrument (Sea-Bird Electronics, Inc.). Continuous recordings at the mooring (“X” Fig. 1) during 2005–2015 of conductivity, temperature and depth in 60 m depth was recorded by SBE-37 instruments. The sensors were factory calibrated prior to each field campaigns. Potential temperature and practical salinity (S_P_, calculated from conductivity (PSS78) and referred to as S in the text) measured between July and August were analyzed from the “Dybet” section (i.e. stations between 21.727 °W and 21.749 °W, Fig. 1) in the period 2005–2015 and an additional profile from March 2005 were obtained from the central “Dybet” section. The bathymetry of the fjord was based on echo soundings and interpolation^27^ and a hypsographic curve was made for the “Dybet” section (Fig. 1).

A Simple box model of the “Dybet” section (Fig. 3) described the conditions below the interface depth (D_i_ = 240 m) and the volume-averaged concentration φ and the corresponding concentration φ* in the layer between D_u_ and D_i_. Integration of Eq. (1) from the bottom to D_i_ results in:

2 $$\frac{\partial \phi }{\partial t}={\eta }^{-1}({k}_{v}\frac{\partial \phi }{\partial z}(z=-{D}_{i})+{F}_{\phi })$$

where η (48.4 m) is the ratio of the volume of the Dybet section (1.08 km^3^) and the horizontal area at D_i_ (22.3 km^2^), and F_φ_ is the bottom flux (i.e. F_S _= 0 for salinity and F_H_ is the GHF of the heat content H = ρ c_p_ θ, where ρ and c_p_ are density and the specific heat capacity of seawater, respectively). By integration the turbulent exchange coefficient across D_i_ is calculated from year to year as:

3 $${k}_{v}=\eta \frac{S({t}_{{\rm{0}}})-S({t}_{{\rm{0}}}-\Delta t)}{\Delta t}{(\frac{\partial S}{\partial z}(z=-{D}_{i}))}^{-1}$$

where the timestep Δt is one year, S(t) is the volume averaged salinity below D_i_ at time t and the vertical salinity gradient is estimated from ∂S/∂z(z = −D_i_) ~ (S^*^ − S)/(D_u_ − D_i_). The GHF is finally calculated from:

4 $${F}_{H}={F}_{Htot}-{F}_{Hmix}=\eta \frac{H({t}_{{\rm{0}}})-H({t}_{{\rm{0}}}-\Delta t)}{\Delta t}-{k}_{v}\rho {c}_{p}\frac{\partial \theta }{\partial z}(z=-{D}_{i})$$

where F_Htot_ is the total heat change and F_Hmix_ is the contribution from vertical exchange across D_i_.

### Data availability statement

The datasets generated during and/or analysed during the current study are available from the corresponding author on reasonable request.
